# Associations between individual factors, environmental factors, and outdoor independence in older adults

**DOI:** 10.1007/s10433-020-00553-y

**Published:** 2020-02-07

**Authors:** Barbara Schehl, Joerg Leukel

**Affiliations:** Institute for Health Care and Public Management, Faculty of Business, Economics and Social Sciences, University of Hohenheim, Schwerzstraße 35, 70599 Stuttgart, Germany

**Keywords:** Outdoor independence, Older adults, Ecological approach, Survey

## Abstract

The degree to which individuals can accomplish outdoor activity by themselves or require support is an important facet of successful aging. While prior research focuses on participation in outdoor activity, understanding of older adults’ outdoor independence is limited. We adopt an ecological approach to examine the role of individual factors and environmental factors in explaining outdoor independence. Our sample comprised older adults aged 65 + living in a medium-sized city in Germany (*N* = 1070). The results show that being male, younger, and healthier was positively associated with outdoor independence, while living together was not. Further, outdoor independence decreased with higher levels of perceived environmental barriers. This negative association was moderated such that it was stronger for the less healthy and older participants. Based on our empirical findings, we offer insights for policy makers, urban planners, and community groups to design age-friendly communities and consequently facilitate outdoor independence among older adults.

## Introduction

In an aging society, remaining independent and maintaining an active lifestyle is important to many older adults. Active behavior can delay aging processes and contribute to successful aging (Diggs 2008). A particular type of activity is outdoor activity such as shopping, visiting friends, and attending social events. Outdoor activity can enhance quality of life (Vagetti et al. 2015) and is perceived as more pleasurable than activity performed at home (Cabrita et al. 2017).

Participation in outdoor activity depends on the environment in which older adults live. In other words, older adults residing in a supportive environment tend to be more active outside (Eronen et al. 2013; Sugiyama and Thompson 2007). The role of the environment in explaining older adults’ outdoor activity has been studied extensively (Moran et al. 2014; Rosso et al. 2011) The overall finding is that various environmental conditions should be considered, e.g., quality of sidewalks, availability of resting places, and access to amenities. While previous research offers important insights into activity patterns of particular subgroups, understanding of older adults’ outdoor independence is limited. Outdoor independence is defined as the degree to which an individual believes in their ability to be active outdoors. For instance, independence can be categorized whether one needs support by others or is able to be active by themselves. However, little is known about the factors affecting outdoor independence. The paucity of prior research is surprising given that independence is an important predictor of many outdoor activities.

To understand how people interact with their environment, ecological models have been found useful (Fisher et al. 2018; Sallis et al. 2008). The main tenet of the ecological approach is that multiple levels of influences, such as intrapersonal, cultural, environmental, and political, explain human behavior. Previous research developed such models for explaining participation in outdoor activity (Cunningham and Michael 2004; Sallis et al. 2006). In a similar vein, our study adopts the ecological perspective to examine the role of individual and environmental factors for outdoor independence in older adults. Understanding of how individuals interact with their environments helps to develop effective approaches to improve behaviors. Hence, environments and policies must be created to facilitate healthful choices, and older adults must be educated and motivated about those choices (Sallis et al. 2008).

With respect to individual factors, previous research provides empirical support for several variables being associated with outdoor activity. For instance, men and women seem to have different opportunities and needs to participate in outdoor activity (Bennett 1998; Lee 2005; Sjögren and Stjernberg 2010), e.g., women reported to feel unsafe going out after dark and fear crime as a barrier to outdoor activity (Eyler et al. 1999). Given that the group of older adults is often characterized by a decline in mobility and mental ability as well as functional impairments (Spirduso et al. 2005), it is not surprising that with increase in age participation in physical outdoor activity decreases (Sjögren and Stjernberg 2010; Wu et al. 2016). Similarly, many older adults are experiencing limitations in physical functioning, which impair them in their mobility and independence to leave the house. Thus, being in poor health undermines participation in outdoor activity (Chaudhury et al. 2016). As many older adults tend to leave the house more often when they have partners or friends who accompany them, it is also reasonable to assess participants’ living situation when considering older adult’s outdoor activity (Chudyk et al. 2017; Sugiyama and Thompson 2007).

With respect to environmental factors, the literature provides a comprehensive account of environmental features, which either support or hinder older adults in remaining independent and active (Kerr et al. 2012; Moran et al. 2014; Rosso et al. 2011). Of particular importance are perceptions of environmental barriers, which have been found to be more related to outdoor activity than the objective environment (Wu et al. 2016). Individuals, who have higher perceptions of environmental barriers, are less likely to be active outdoors. For instance, bad signage or missing signs can impede individuals to cross streets, which specifically affect older adults with declining sense of vision and navigation (Chaudhury et al. 2016). Further, dangerous sidewalks enhance the risk of falling due to uneven conditions, steepness, and high curbs (Rosenberg et al. 2013). Similarly, poor lighting of sidewalks has been found to increase the danger of falling (Rosenberg et al. 2013). As older adults often depend on places to sit and rest due to diminishing physical fitness, lack of resting places hinders older adults to leave the house and therefore are perceived as high barrier for outdoor activity (Chaudhury et al. 2012; Moran et al. 2014). Likewise, lack of public toilets is a severe problem for older adult’s outdoor mobility, especially for those suffering from incontinence (Moran et al. 2014; Risser et al. 2010). Another barrier identified in the literature is the distance to essential destinations such as groceries and pharmacies (Rantakokko et al. 2014; Nathan et al. 2014). Next to these factors, participation in outdoor activity can also depend on whether older adults live in the city center or in a peripheral area (Krogstad et al. 2015).

In summary, the ecological approach provides a rationale for examining how individual and environmental factors are associated with outdoor independence. This approach allows us to assess the strength of these factors and their interplay. Specifically, the purpose of our study is to investigate individual and environmental factors related to outdoor independence in older adults. We assess the role of gender, age, subjective health, living arrangement, neighborhood, and perceptions of environmental barriers. The results of our study will inform the planning of age-friendly communities and have policy implications for facilitating sense of outdoor independence among older adults.

## Methods

### Study design and participants

Our study was based on a cross-sectional survey that we conducted in the summer of 2017. This survey targeted all older adults (65 +) living in three neighborhoods of a medium-sized city in Germany. While two neighborhoods exhibited high population density (6056 and 2400 per square kilometer, respectively), the third neighborhood had a clearly rural character and its population density was much lower (343 per square kilometer). A local municipal provider of geriatric care was involved in the survey, in particular by pretesting the questionnaire and implementing the survey. Further, we received support from the city administration, which provided us with the registered addresses. The paper-based questionnaire was mailed to 6170 older adults. It was complemented by a cover letter signed by the respective district leader. We received 1302 valid responses within 6 weeks (response rate: 21.5%, considering that 100 addresses were invalid). This rate is comparable to prior surveys that used posted self-administered questionnaires (Palonen et al. 2016). Participants also had the option to fill in the questionnaire online by using an individual access code; this option was chosen by thirty-six participants.

The current study includes 1070 participants, who answered questions on socio-ecological factors and outdoor independence (no missing values). We evaluated whether our convenient sample is representative of the population as a whole. Specifically, we compared our sample with the population of older adults living in the city from which the sample was drawn. We retrieved data from a database provided by the state (IT.NRW 2017). The share of women in our sample (50.0%) and the population (51.0%) was very similar. There were only marginal differences with respect to age groups, categorized into 65–69 years (25.5% vs. 25.7%), 70–79 years (45.5% vs. 46.9%), and 80 years and older (29.0% vs. 27.4%). The share of participants with no high school education was smaller in our sample (1.1% vs. 6.1%), while the share of older adults holding an academic degree was greater (14.7% vs. 6.5%).

### Measurements

The study examined the role of individual and environmental factors associated with outdoor independence. Individual factors included gender, age, subjective health, and living arrangement. The main environmental factor under study was perceived environmental barriers. In addition, we controlled for neighborhood.

### Outdoor independence

O*utdoor independence* was measured by asking participants: “Which of the following activities do you accomplish on your own, for which do you need support?”. We chose shopping, visiting doctors, attending events, and visiting friends as activities being most prevalent in older adults (Szanton et al. 2015). We administered a four-point scale ranging from “not possible even with support” (0), “with personal support only” (1), and “independent only on known routes” (2) to “independent (without support)” (3). The answers were summed to produce an independence score ranging from zero to twelve. Scale reliability was excellent (Cronbach’s alpha of .94). The scores were then mapped onto four groups indicating the level of outdoor independence, defined as very low for 0–3, low for 4–7, medium for 8–11, and high for 12.

### Individual factors

*Gender* was defined as female or male. *Age* was calculated based on participant’s year of birth. *Subjective health* was operationalized by perceptions of one’s individual health. We defined the question as follows: “How satisfied are you with your health state?”. Answer options ranged from “very bad” (1), “rather bad” (2), “moderate” (3), and “rather good” (4) to “very good” (5) (Idler and Benyamini 1997). *Living together* was assessed by asking participants about the number of people living in their household. Then, we derived a dichotomous variable (yes or no) (Chudyk et al. 2017; Sugiyama and Thompson 2007).

### Environmental factors

The main environmental factor in our study was *perceived barriers* defined as perceptions of how far the built environment hinders one’s outdoor activity (Rantakokko et al. 2017; Sugiyama and Thompson 2007). We operationalized this factor by asking: “To what extent do the following circumstances prevent you from going outdoors?”. We defined a six-item instrument based on prior research (Moran et al. 2014; Rosso et al. 2011): Bad signage/missing signs; dangerous sidewalks; poor lighting of sidewalks; lack of resting places; lack of public toilets; long distances. Participants evaluated the relevance of each barrier using a five-point scale (Michael et al. 2006). The scale ranged from “not at all” (1), “somehow” (2), “moderately” (3), and “strongly” (4) to “very strongly” (5). Finally, we calculated the mean of the six items. Therefore, perceived barriers were defined as a continuous variable. Cronbach’s alpha of our instrument was .86, which indicates a good level of internal consistency. In addition, we controlled for *neighborhood* (urban or rural) (Krogstad et al. 2015).

### Statistical analyses

Descriptive statistics included the mean, standard deviation, minimum, maximum, and frequencies. Bivariate correlations for all variables were assessed by using the Spearman correlation test (no variable was normally distributed). Because our dependent variable was measured on an ordinal scale with four categories, we conducted ordinal regression analysis. This analysis determined odds ratios (OR), which represent how the probability of achieving a higher level of outdoor independence changes for one-unit increase in the independent variable (OR > 1 for positive changes, OR < 1 for negative changes). We followed a hierarchical approach: While the first model only comprised individual factors, the second model also included environmental factors. The third model added four interaction terms for individual factors and perceived barriers, which allowed us to examine moderation. We mean-centered all metric variables to assist in interpreting the model with interactions by giving age, subjective health, and barriers meaningful zero-points (Dalal and Zickar 2012). For each model, we tested the assumptions of ordinal regression analysis, in particular, no multicollinearity and proportional odds. Multicollinearity was assessed using variance inflation factors (VIF), which were between 1.07 and 1.33. This result showed that multicollinearity did not affect our regression models (Hair et al. 2014). The proportional odds assumption was verified by using the parallel lines test for each regression model. The *p* values were between .170 and .423; hence, the proportional odds assumption was met, meaning that the slope of regression lines is the same for each level of outdoor independence (Garson 2012). All statistical analyses were carried out using IBM SPSS Statistics 25. The significance level was 5%.

## Results

Table 1 shows descriptive statistics for the independent variables. The sample was balanced in terms of gender. Almost half of the participants were between 65 and 74 years (46.0%), while 11.5% were older than 84 years. With respect to subjective health, good/very good accounted for 52.2%, and bad/very bad was reported by 10.4%. More than two-thirds of the participants lived with someone (70.7%). About every seventh reported that environmental barriers did not at all prevent them from going outdoors (16.7%), while 19.2% had strong or very strong perceptions of environmental barriers. The number of participants per neighborhood was similar, with two-thirds living in the urban neighborhoods.

**Table 1:** Participant characteristics, *N* = 1070

Variable	*n*	(%)	*M*	(SD)	Range
Gender					
Male	535	(50.0)			
Female	535	(50.0)			
Age (years)^a^			74.99	(6.93)	65–98
65–74	483	(46.0)			
75–84	448	(42.6)			
85–94	112	(10.7)			
≥ 95	8	(.8)			
Subjective health (1–5)			3.52	(.88)	1–5
Very bad	20	(1.9)			
Bad	91	(8.5)			
Moderate	401	(37.5)			
Good	433	(40.5)			
Very good	125	(11.7)			
Living together					
Yes	757	(70.7)			
No	313	(29.3)			
Barriers (1–5; b)^b^			2.14	(.96)	1–5
1	179	(16.7)			
1 < b ≤ 2	408	(38.1)			
2 < b ≤ 3	294	(27.5)			
3 < b ≤ 4	173	(16.2)			
4 < b ≤ 5	32	(3.0)			
Neighborhood					
Urban	728	(68.0)			
Rural	342	(32.0)			

^a^We defined four age groups for the sake of clarity

^b^Higher scores indicate higher levels of perceived environmental barriers

With respect to outdoor independence, the frequencies for the levels were as follows: Very low independence was reported by 44 participants (4.1%) and low independence by 98 (9.2%). Also, 98 older adults indicated a medium level (9.2%) and 830 stated high independence (77.6%).

Table 2 presents the correlation matrix. For all independent variables, most correlations were weak, and the correlation between subjective health and perceived barriers was moderate (*r* = − .42). Correlations between predictors and outdoor independence were statistically significant, with independence decreasing for participants being female (*r* = − .16), older (*r* = − .43), and reporting higher perceived barriers (*r* = − .42). Outdoor independence was higher for those who were healthier (*r* = .47) and lived together (*r* = .20). Neighborhood was not correlated with outdoor independence (*r* = − .01, *p* = .88).

**Table 2:** Correlations for predictors and outdoor independence, *N* = 1070

	1.	2.	3.	4.	5.	6.
1. Gender (female)						
2. Age	.08**					
3. Subjective health	− .07*	− .28**				
4. Living together	− .22**	− .24**	.12**			
5. Barriers	.14**	.31**	− .42**	− .19**		
6. Neighborhood	.02	− .02	− .03	− .09**	.01	
7. Outdoor independence	− .16**	− .43**	.47**	.20**	− .42**	− .01

Spearman’s rank correlations (two-tailed). *p < .05, **p < .01

Table 3 provides the results of the ordinal regression analyses. Model 1 includes the four individual factors, of which gender, age, and subjective health were associated with outdoor independence. By adding environmental factors, Model 2 shows that women were less likely to be independent outdoors (OR = .65). The probability for the next level of outdoor independence was 12% lower for each one-year increase in age (OR = .88). This probability was 185% higher for each one-unit increase in subjective health (OR = 2.85). Living together was not associated with outdoor independence. Concerning the environmental factors, perceived barriers exhibited a negative association (OR = .57) and this association remained after controlling for neighborhood. Adding environmental factors to the regression model led to an increase of explained variance by 7% (*R*^*2*^ = .54), which suggests that individual factors and environmental factors worked in concert. To further explore this finding, we assessed interactions between individual factors and environmental barriers.

**Table 3:** Associations between individual factors, environmental factors and outdoor independence (ordinal regression models), *N* = 1070

Variable	Model 1 individual factors			Model 2 environmental factors added			Model 3 interaction terms added		
	OR	95% CI	*p*	OR	95% CI	*p*	OR	95% CI	*p*
Gender (female)	.61	.46–.80	**< .001**	.65	.49–.86	**.003**	.74	.56–.97	**.028**
Age (years)	.87	.86–.89	**< .001**	.88	.86–.89	**<.001**	.91	.89–.93	**< .001**
Subjective health (1–5)	3.17	2.70–3.72	**< .001**	2.85	2.41–3.36	**<.001**	2.12	1.81–2.49	**< .001**
Living together (yes)	.83	.63–1.09	.177	1.03	.78–1.36	.842	.91	.68–1.23	.540
Barriers (1–5)				.57	.49–.65	**<.001**	.77	.61–.97	**.029**
Neighborhood (urban)				.99	.75–1.32	.954	1.00	.77–1.29	.977
Barriers × Gender							.83	.64–1.07	.154
Barriers × Age							.98	.96–1.00	**.040**
Barriers × Subjective health							1.38	1.18–1.62	**< .001**
Barriers × Living together							1.06	.81–1.38	.686
*R*^2^	.47			.54			.66		
*R*^2^ change	n/a			.07			.12		

Bold values show significance

Age, subjective health, and barriers were mean-centered

Link function: complementary Log–log

*OR* odds ratio, *CI* confidence interval, *R*^2^ pseudo *r*-squared (Nagelkerke’s)

As the results of Model 3 indicate, the interaction between environmental barriers and subjective health was statistically significant, i.e., the negative association of perceived environmental barriers was stronger for the less healthy (OR = 1.38). Further, the role of perceived barriers was smaller for younger participants (OR = .98). Subsequently, inserting interaction terms further increased the explanatory power to 66%. Gender, age, subjective health, and barriers remained statistically significant in Model 3. We note that each reported OR can only be interpreted as the unique impact on outdoor independence when all other mean-centered variables are zero.

## Discussion

This study investigated individual and environmental factors linked to outdoor independence in older adults. The ecological approach offered a structure for investigating this link. Our study is first in testing the role of individual factors in explaining outdoor independence and thus contributes to understanding this important facet of active aging. In combination with environmental factors, our model is effective by explaining two-thirds of the variance in outdoor independence. Overall, our study results suggest that both individual and environmental factors should be considered to understand outdoor independence.

With respect to individual factors, we found that being male, younger, and healthier was positively associated with outdoor independence. Previous studies have shown that individual factors are similarly associated with participation in outdoor activity, either measured by duration or frequency. For instance, gender differences were found for participation in both light and moderate-to-vigorous physical activity, with lower levels of participation for women but not in case of walking for transportation (Chudyk et al. 2017). Higher age also undermined participation, e.g., in terms of total activity count, steps taken per day, levels of physical activity (Chudyk et al. 2017), and outdoor recreational activity (Wu et al. 2016). Results of previous research are consistent for the positive role of health for participation in outdoor activities (Sugiyama and Thompson 2007). Prior findings for the role of social factors such as living arrangement, marital state, and social network are less conclusive (van Holle et al. 2015). In our sample, living together with someone did not enhance the odds of outdoor independence. An explanation for this result may be that our measure did not reflect the extent to which one receives social support by the partner or family (Carlson et al. 2012; Corseuil Giehl et al. 2017); hence, specific types of social support may also facilitate outdoor independence.

Our results underscore the relevance of the environment such that when supportive features exist, older adults can remain independent and active (Kerr et al. 2012). Environmental barriers have been studied extensively in the older adults’ outdoor activity literature (Moran et al. 2014; Rosso et al. 2011). While previous studies showed a negative association between perceived environmental barriers and outdoor activity, we find a similar association for outdoor independence. Moreover, the combination of perceived barriers and individual factors should be considered when examining outdoor independence. By analyzing whether individual factors moderate the influence of perceived environmental barriers, we treated the older population as a diverse group. We find that the negative association of environmental barriers was stronger for less healthy and older participants. Aging is often accompanied with deteriorating mobility, physical fitness, and mental abilities, which culminate in lower levels of subjective health. These inevitable developments make older adults feel intimidated and unsafe, thus, impeding them to leave the house. Consequently, those less healthy older adults might have lower levels of confidence in their ability to be active outdoors. While prior research tested interaction effects derived from ecological models, few studies found such interactions (Carlson et al. 2012; Slaug et al. 2019; van Holle et al. 2015). In our context of outdoor independence, the results demonstrate subjective health and age as individual factors, and perceived barriers as an environmental factor work in concert; hence, together they even have a stronger impact.

Collectively, our study results provide strong evidence for the usefulness of the ecological approach to explain outdoor independence. Our study complements previous research that focused on participation in outdoor activity. The usefulness lies in being able to assess the role of individual factors, environmental factors, and their interactions. Indeed, we find two meaningful interactions between subjective health, age, and environmental barriers, which further enhance the explanatory power of our model.

Two constructs related to outdoor independence can be identified from the extant literature, namely mobility and autonomy. Mobility consistently emerges as an important facet of older adults’ outdoor activity. As outdoor mobility is connected to many necessary daily activities as well as leisure activities, it is not surprising that a decline in outdoor mobility is associated with lower levels of quality of life (Rantakokko et al. 2014). Previous research showed that barriers to outdoor mobility can be attributed to personal, social, environmental, technical, and legal aspects (Risser et al. 2010). Particularly, older adults rated inconsiderate car drivers and lack of public toilets as the most important barriers to outdoor mobility. This issue gained increasing prominence on a European level. For instance, the European Union’s project SIZE (Life quality of senior citizens in relation to mobility conditions) focused on older adults’ mobility and transport situation as well as experts’ views on this (Amann et al. 2006). Specifically, the results of SIZE provide guidance for implementing relevant policies to maintain older adult‘s mobility. Although mobility is important for older adults’ outdoor activity, we note that outdoor mobility must not be confused with outdoor independence. While mobility is defined as the ability to move easily outside, our study examined whether older adults can accomplish outdoor activities with or without support.

Autonomy occurs as another construct in the context of outdoor activity. Several studies applied the Impact on Participation and Autonomy Questionnaire (IPAQ) (Cardol et al. 2001) to assess autonomy in participation outdoors (Rantakokko et al. 2017; Portegijs et al. 2014). It is worth noting that the conceptualization of autonomy in previous research is different from outdoor independence. Autonomy represents participation in outdoor activity, but does not differentiate whether one needs support. In our study, we examined whether older adults require support by others or can be active outdoors by themselves.

We believe that our findings have three practical implications. First, our results help identify subgroups requiring support most urgently. One particular group is older women. Our results suggest that women are at higher risk of losing their feeling of outdoor independence. Therefore, appropriate activity promotion strategies are needed, tailored to the needs of women. Second, understanding the predictors of outdoor independence can assist policy makers, urban planners, and community groups in designing interventions targeted to the needs of older adults. The perceived environmental barriers can be addressed by, e.g., barrier-free signs to increase orientation, safe sidewalks, improved lightning of sidewalks as well as shorter distances to resting places, public toilets, and amenities. Such environmental components can be implemented by municipalities, local businesses, and civil associations. By transforming the infrastructure into an age-friendly and barrier-free environment, feelings of independence will be strengthened, and ultimately, participation in outdoor activity will be enhanced. Third, older adults’ outdoor independence can also be facilitated by providing services that arrange companionship. Implementing and maintaining such social interventions would require less resources than environmental interventions, especially when considering the severely limited public budgets.

The results of this study should be interpreted in light of its limitations. As it is the nature of surveys using self-reports, participants’ overestimation of outdoor independence might be possible; hence, our data are subjective and rather approximate. While our study focuses on a core set of individual and environmental factors, future research can examine the usefulness of further factors. Of particular interest are mobility (Risser et al. 2010) and social support ((Fisher et al. 2018), which are both essential for this target group. Fellow researchers could also consider objective measures of the environment, which can be retrieved by GIS databases (Nathan et al. 2014; Wu et al. 2016).

## Conclusion

The contribution of this study is an empirically validated model explaining outdoor independence through individual and environmental factors. Using an ecological approach, our study results suggest that the immediate environment is an essential attribute to understand older adults’ outdoor independence. The perceptions of how far the built environment hinders one’s activity largely determine outdoor independence. Further, our analysis revealed that being female, older, and less healthy enhanced the risk for low levels of outdoor independence. However, outdoor independence was not associated with living together. Perceived environmental barriers were more important for those who reported lower levels of health and were older. This result indicates that environmental interventions should target less healthy older adults, because behavioral changes can most likely be expected in this subgroup. Collectively, our findings contribute a profound understanding of factors associated with outdoor independence. Specifically, our research provides the foundation for deeper inquiry as signified by our model explaining about two-thirds of the variance. We believe that our results offer insights for policy makers, urban planners, and community groups to design age-friendly communities, which then ultimately can facilitate outdoor independence among older adults.
